# When one door closes: a qualitative exploration of women’s experiences of access to sexual and reproductive health services during the COVID-19 lockdown in Nigeria

**DOI:** 10.1186/s12889-023-15848-9

**Published:** 2024-04-23

**Authors:** Babatunde Adelekan, Lanre Ikuteyijo, Erika Goldson, Zubaida Abubakar, Oluwatomi Adepoju, Olaitan Oyedun, Gbenga Adebayo, Andat Dasogot, Ulla Mueller, Adesegun O. Fatusi

**Affiliations:** 1 United Nations Population Fund (UNFPA) Country Office, Abuja, Nigeria; 2 Department of Sociology and Anthropology, Faculty of Social Sciences, Obafemi Awolowo University, Ile-Ife, Nigeria; 3 Academy for Health Development (AHEAD), Ile-Ife, Nigeria; 4 Livinghealth International, Lagos, Nigeria; 5 Centre for Adolescent Health and Development, School of Public Health, University of Medical Sciences, Ondo, Nigeria; 6 Department of Community Health, Faculty of Clinical Sciences, College of Health Sciences, Obafemi Awolowo University, Ile-Ife, Nigeria

**Keywords:** COVID-19, COVID-19 lockdown, Sexual and Reproductive Health, Sexual and Reproductive Health Services, Healthcare Access

## Abstract

**Background:**

COVID-19 pandemic widely disrupted health services provision, especially during the lockdown period, with females disproportionately affected. Very little is known about alternative healthcare sources used by women when access to conventional health services became challenging. This study examined the experiences of women and adolescent girls regarding access to sexual and reproductive health (SRH) services during the COVID-19 lockdown in Nigeria and their choices of alternative healthcare sources.

**Methods:**

The study sites were two northern states, two southern states, and the Federal Capital Territory. Qualitative data were obtained through 10 focus group discussion sessions held with married adolescents, unmarried adolescents, and older women of reproductive age. The data were transcribed verbatim and analysed using a thematic approach and with the aid of Atlas ti software.

**Results:**

Women reported that access to family planning services was the most affected SRH services during the COVID-19 lockdown. Several barriers to accessing SRH services during COVID-19 lockdown were reported, including restriction of vehicular movement, harassment by law enforcement officers, fear of contracting COVID-19 from health facilities, and fear of undergoing compulsory COVID-19 tests when seeking care in health facilities. In the face of constrained access to SRH services in public sector facilities during the COVID-19 lockdown, women sought care from several alternative sources, mostly locally available and informal services, including medicine vendors, traditional birth attendants, and neighbours with some health experience. Women also widely engaged in self-medication, using both orthodox drugs and non-orthodox preparations like herbs. The lockdown negatively impacted on women’s SRH, with increased incidence of sexual- and gender-based violence, unplanned pregnancy resulting from lack of access to contraceptives, and early marriage involving adolescents with unplanned pregnancies.

**Conclusion:**

COVID-19 negatively impacted access to SRH services and forced women to utilise mostly informal service outlets and home remedies as alternatives to conventional health services. There is a need to ensure the continuity of essential SRH services during future lockdowns occasioned by disease outbreaks. Also, community systems strengthening that ensures effective community-based health services, empowered community resource persons, and health-literate populations are imperative for overcoming barriers to healthcare access during future lockdowns.

## Background

The World Health Organization, on the 30th of January 2020, declared the outbreak of the novel human coronavirus disease 2019 (COVID-19) as a Public Health Emergency of International Concern [1]. With this declaration, the infection, which was first reported in Wuhan, China, in December 2019, became formally recognised as “an extraordinary event that may constitute a public health risk to other countries through the international spread of disease and may require an international coordinated response” [1]. The declaration, thus, sounded the wake-up call for galvanising actions on a global scale towards controlling the then fast-spreading infection. With the non-availability of effective drugs or vaccines in the early phase of the pandemic, countries responded with a variety of non-pharmaceutical public health control measures, of which “stay at home” orders or “lockdown” featured prominently in many settings [2–4]. Several countries ordered a lockdown at the national or sub-national level aiming to reduce contact among individuals and between population groups and thereby slow the rate of spread of COVID-19 [2–4].

The federal government of Nigeria ordered the first nationwide lockdown on the 30th of March 2020 [5, 6], by which time the country had recorded 133 confirmed cases of COVID-19 spread across 12 of its 36 states [5, 7]. The lockdown order required people to stay at home while businesses, schools and recreational facilities were shut down, social and religious gatherings curtailed, intra-city and intra-state movements were restrained, and inter-city travels banned except for some essential groups of workers. The lockdown was for an initial period of two weeks but was later extended for another three weeks [5, 6]. By 27th February 2022, two years after its index case was recorded, Nigeria had recorded 254,525 confirmed cases of COVID-19 and 3142 deaths [7].

Studies have shown that while the lockdown measures contributed positively towards reducing the rate of spread of COVID-19 infection, it negatively impacted the availability as well as the accessibility of health services in many settings, with women and young people in low- and middle-income countries disproportionately affected [8–10]. As the Guttmacher-Lancet Commission has noted, “Sexual and reproductive health and rights are fundamental to people’s health and survival, to economic development, and to the wellbeing of humanity” [11]. Access to quality sexual and reproductive health (SRH) services plays a vital role in the well-being of a human being, and essential SRH services include contraceptive services; maternal and newborn care; prevention and treatment of HIV/AIDS; control and care for other sexually transmitted infections other than HIV; safe abortion care; prevention, detection and counselling for gender-based violence; and, counselling and care for sexual health and well-being [11, 12]. Women are particularly at risk of negative SRH outcomes like unplanned pregnancy, maternal morbidity, and maternal mortality when there is reduced access to family planning and skilled birth attendants [11]. Thus, there is the need to maintain access to quality SRH services, even in the face of a pandemic like COVID-19 and containment measures like lockdown [13]. The need for quality and accessible SRH services further increases during the lockdown period as researchers have documented an increased rate of sexual and gender-based violence (SGBV) in that context [14, 15].

Yet, research findings from Nigeria [16–18] and elsewhere [19–23] indicate that COVID-19 and the associated lockdown negatively affected access to SRH services. The reported impact varied considerably by geographies, social and health services contexts, and population settings. Overall, however, there are still significant gaps in knowledge regarding the effect of COVID-19 and lockdown on access to health services [24], and SRH services specifically [12, 14]. A recent scoping review – published in the first quarter of 2022 – specifically noted that “qualitative studies available up to date are still limited in both number and scope” [24] regarding the impact of COVID-19 on healthcare access. Another major gap in the existing literature is that little is known regarding the alternate sources of SRH care used by women in the face of challenges with accessing orthodox health services. Furthermore, while Nigeria is a multi-ethnic country with significant regional differences in health-related behaviours and associated factors, studies regarding COVID-19 and healthcare access have been very limited in their geographic focus. Except for the quantitative study by Adelekan and colleagues [16], studies on COVID-19 and access to healthcare in Nigeria have generally been limited to only one of the country’s 36 states [17–18].

Nested within a research work that spanned both the southern and northern parts of Nigeria, we specifically address two key gaps identified above, which have not been satisfactorily addressed in previous studies [10–16]. First, the effect of the COVID-19 lockdown on women’s access to SRH care; and, secondly, the healthcare sources that women used for SRH care when services could not be accessed from the public sector health facilities. Access to health care is a multidimensional concept [25, 26]: we adopted the model of Rodriguez Santana et al. as our framework of reference in examining access to SRH services. This model conceptualizes access as consisting of three elements: (1) service availability (having access); (2) levels of utilisation and barriers to utilisation (getting access) that are both structural (supply-side) and individual-specific (demand side); and (3) the effectiveness of health care supply in terms of how well it aligns with needs [26].

## Methods

### Study design, setting, and data collection

This qualitative study is part of a larger research initiative with mixed methods of data collection to assess the impact of an intervention project aimed at improving access to essential health services during COVID-19. The project was implemented in the Federal Capital Territory (FCT) and nine of Nigeria’s 36 states from June 2020 to 31 May 2021. This article is based on the qualitative data collected through in-person focus group discussion (FGD) with women of reproductive age over 10 days in June 2021 as part of the project evaluation. Specifically, this qualitative study was implemented in five geographic locations: the Federal Capital Territory, Abuja; two states in the northern part of Nigeria (Kaduna and Borno); and two states in the southern part of Nigeria (Akwa Ibom and Lagos). The sampling technique took into consideration the project-related health facilities in the intervention locations/states.

We undertook 10 FGD sessions with purposively-selected women of reproductive age, consisting of both married and unmarried adolescents (aged 15–19 years) and older women (aged 20–49 years). Our inclusion criteria included residence in the focal communities during the period spanning the COVID-19 lockdown and project intervention (March 2020 to 31 May 2021), and good knowledge of women’s SRH care-seeking experiences during the lockdown. Those not willing to voluntarily give consent and healthcare workers – both orthodox and non-orthodox (including traditional birth attendants) – were excluded from the study. Each FGD session was conducted face-to-face and included five participants per session to allow for adequate social distancing in line with the COVID-19 protocol approved then by the government. Each FGD session lasted an average of one hour. The FGD sessions were facilitated by experienced research personnel who were familiar with the norms and values of the study communities and supported by note-takers who took relevant notes by hand as well as audio-recorded the FGD sessions. English language, which is Nigeria’s official language, was the primary language of communication for the FGDs. However, indigenous Nigerian languages were also used to facilitate the FGD discussions in some communities where the majority of the participants could not communicate effectively in the English language.

We developed the interview guides for the study based on the research objectives and in the context of our adopted framework on healthcare access with a focus to interrogate the experiences of women regarding access to, and use of SRH services during the COVID-19 lockdown [26]. The guide also captured the issue of acceptability, given the growing idea that the accessibility of health care must be accompanied by high levels of acceptability [27]. Framed in the context of the COVID-19 lockdown and the access of women to SRH care, the main questions focused on the degree to which SRH care was accessible from formal healthcare sources, particularly public sector facilities; constraints and barriers to accessing care; alternative healthcare sources utilised for SRH care, and factors that influenced choices of SRH care sources. The questions were mostly unstructured, and allowed free-flowing discussion among participants and narration of experiences, as well as probing by the research personnel to explore any area of discussion more deeply and gain greater insights into the participants’ accounts.

### Data analysis

For the FGD sessions that were conducted in the English language, the audio-recorded data were transcribed in that language. However, for the sessions that were conducted in indigenous Nigerian languages, the audio-recorded FGD sessions were first transcribed in the indigenous languages, followed by a translation into the English Language. To ensure rigour and trustworthiness of the data, research assistants who conducted the interviews were involved in the transcription. and the transcripts were randomly checked for accuracy via a process of peer review. The peer-review process entailed the cross-checking of the transcripts by transcribers located in another state.

For data analysis, we used ATLAS.ti software and thematic approach. We consider the thematic approach suitable, given the nature of the study objectives and the consequent exploration of the views, opinions and experiences of women on SRH services. Also, the thematic analysis approach has the merit of enabling the creation of a logical structure for the research, especially as guided by the study objectives [28]. The data analysis was conducted in line with the steps outlined by Braun and Clarke [28], and Silverman [29]. The process started with the data analysts reading the transcribed materials to get familiarised with and make sense of the data. Thereafter, initial codes were generated through both deductive and inductive coding. The deductive codes were generated with the aid of a codebook that was developed by a team of analysts. The codes were developed into themes and the themes formed the basis for report writing. To ensure inter-coder reliability, the coding was done by two analysts as guided by the codebook and emerging codes were flagged and discussed to avoid arbitrariness in the process of coding all the transcripts [29, 30].

### Ethical considerations

We obtained the ethical approval for the study from the National Health Research Ethics Committee domiciled at the Federal Ministry of Health, Abuja. Before the commencement of each FGD session, we obtained informed consent from all the participants. in line with the Nigerian Code of Health Research Ethics [31], we obtained the informed consent of parents or legal guardians as well as the assent of the participants for unmarried adolescents aged less than 18 years. Among others, we informed the women that participation in the study was completely voluntary and they were not obligated to answer any question they did not feel like answering, The women were also informed that if they chose to participate, they are at liberty to discontinue participation at any time. We assured the eligible women that their failure to participate and whatever responses they provide would not have any implications regarding the types and quality of care they could receive in the future. We also assured participants of confidentiality – that all the data generated from the discussions will be completely anonymised. Finally, we ensured that the participants had no relationships whatsoever with the interviewers (either as patients or relatives).

## Results

A total of 94 women of reproductive age participated in the FGD sessions across five states. Adolescents constituted almost half of the study population (48.9%), while never-married women constituted about two-thirds (59.1%) and the majority professed Christianity (67.2%) (Table 1).

**Table 1:** Sociodemographic characteristics of FGD participants

	Akwa Ibom	Borno	FCT	Kaduna	Lagos	Total	
	N = 20	N = 20	N = 19	N = 20	N = 15	n	%
**Age**							
15–19 years	10	10	11	9	6	46	48.9
≥ 20 years	10	10	08	11	9	48	51.1
*Total*						*94*	*100*
**Marital status**							
Never married	12	11	12	9	11	55	59.1
Ever married	8	9	06	11	4	38	41.9
*Total*						*93*	*100*
**Religion**							
Christianity	20	0	20	9	15	64	67.4
Islam	0	20	0	11	0	31	32.6
*Total*						95	100

The key themes that emerged from the study are: (i) the availability of SRH services during COVID-19 lockdown and service gaps for women needing SRH care; (ii) the challenges faced by women in accessing SRH services during COVID-19; (iii) the healthcare sources utilised by women for SRH care when faced with barriers to accessing care from public facilities during COVID-19 lockdown; (iv) factors influencing the choice of alternative sources of SRH care by women, (iv) the effect of the barriers to SRH healthcare access on women’s health. Our findings are broadly organised based on the adopted healthcare access framework of Rodriguez Santana and colleagues [26].

### Availability of SRH services during the COVID-19 lockdown

Participants reported that family planning service was the most affected in terms of SRH service availability during the COVID-19-associated lockdown period, followed by ante-natal and post-natal services. The major factors associated with the restricted access to services included the closure of some facilities and the reduced availability of staff at many facilities due to the social distancing protocol adopted at the facilities. The reduction in the number of staff available at any point in time in the facility translated to a reduction in the number of clients that could use the facilities at a time. Thus, many of the participants had to visit the facilities repeatedly before they obtain the desired services. The situation made some clients seek alternative sources of care.

*Hospitals were not open; they were restricted. Like until when you are severely sick, that’s when you can go to the hospital (****Adolescents’ Group, Akwa Ibom State****).*

*We encountered so many problems during COVID-19 especially regarding antenatal. Before, if you go, the hospitals usually attend to more people and less during the pandemic. For instance, if they attend to 100 people a day, they reduced it to 50 and social distancing must be maintained. As a result, you must visit the hospital for like 3 times before you are being attended to****(Older Women’s Group, Borno State)***

### Getting access to SRH services during the COVID-19 lockdown

**Fig. 1:**
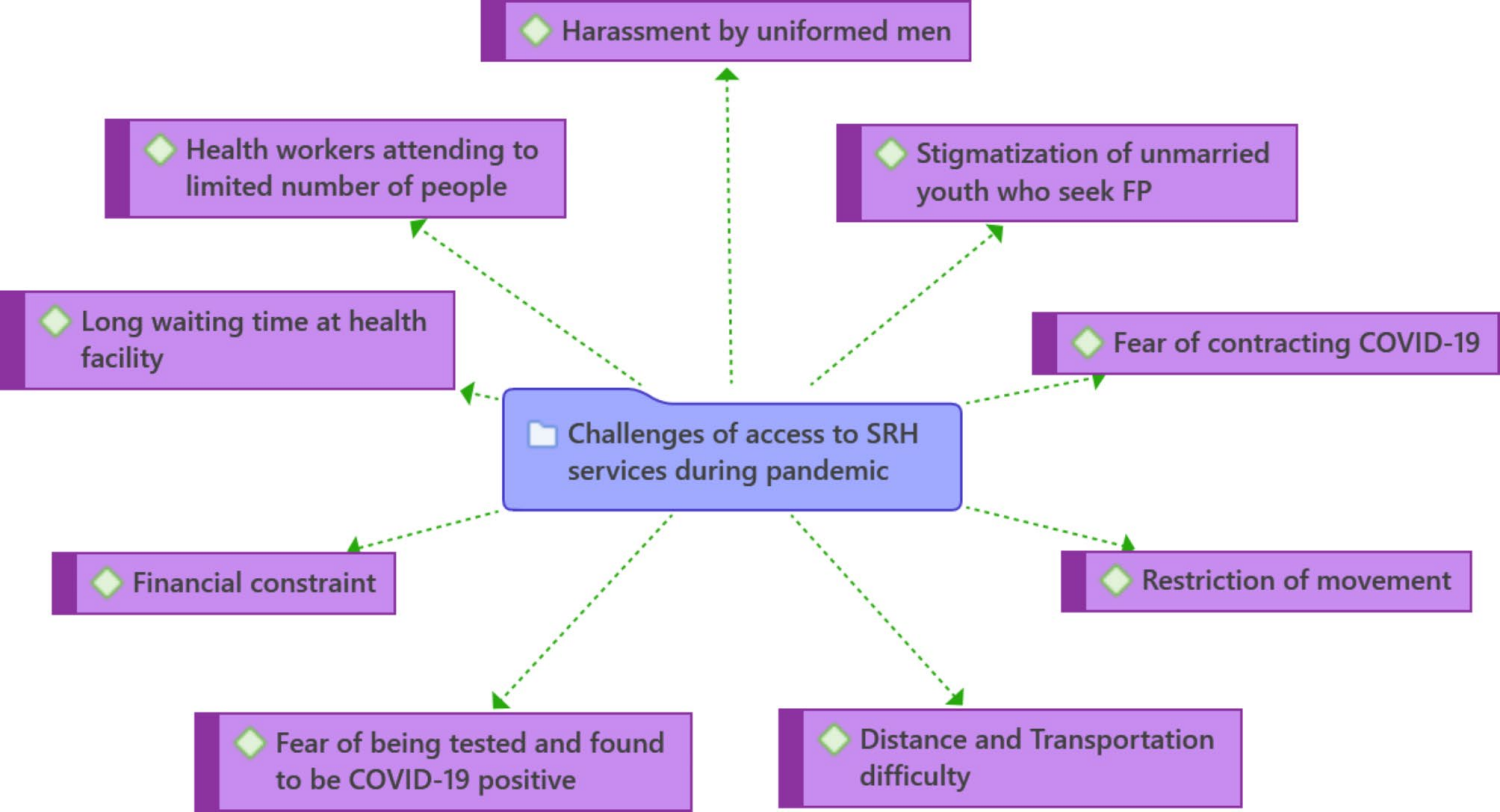
Barriers to accessing SRH services during the COVID-19 lockdown Note: FP = Family Planning, SRH = Sexual and Reproductive Health

Study participants reported encountering several barriers in accessing health services during the COVID-19-associated lockdown (Fig. 1).

Among others, FGD participants, particularly the adolescents (both married and unmarried groups), indicated that some pregnant women could not have access to health facilities due to the challenge of movement restrictions. On the one hand, commercial vehicles were generally not available during the lockdown period. This challenge particularly affected those who did not own personal or family vehicles and those with residences located in places far from health facilities. Many women who needed to use the health facilities were reportedly stranded due to reduced vehicular movements.

*It wasn’t easy because of the restriction in movement. So, there was no vehicle unless there was a private car, then the movement will be easy for you. But still, they will trek (i.e. walk), but as a pregnant woman, I know it won’t be easy for them****(Adolescents’ Group, Abuja).***

*We don’t even get commercial vehicles to convey us. In fact, there wasn’t any means of movement within the town, unless for those that have personal cars and have permit card which is being given by government to some people.****(Older Women’s Group, Borno State).***

On the other hand, in most study states, women recounted experiences of harassment by some law enforcement officers, who prevented them from accessing health facilities and “*some people were even arrested for being on the road”* ***(Adolescent Group, Lagos State).*** Some participants indicated that the presence of police officers on the road and the potential for harassment generally deterred movement and discouraged some women from attempting to go to the health facility in the first instance.

*I kept going to the hospital and sometimes we meet Police officers on the road and if I forget my facemask, they will ask me to go back that I cannot pass. Then we will buy it (face masks) on the road and wear them….I finally gave birth during that Corona period. (****Adolescents’ Group, Akwa Ibom State****).*

The participants, however, also generally indicated that police officers allowed movement when the need for healthcare is obvious, such as in the case of pregnant women.

*There were police on the road but they cannot expect you to just sit down in your room and die there with whatever is wrong with you. They have already seen you, the person with what she is. For example, let us say she is a pregnant woman and she had already told them that they are going to the hospital or maternity, of course, they would allow her. It is just that there may be the problem of transportation (****Older Women’s Group, Lagos State)***

Another barrier reported by women regarding access to maternal health services was financial constraints as economic activities were practically at a standstill during the COVID-19 lockdown.

Most pregnant women could not afford to pay for some maternal health services in orthodox healthcare settings, and some reported being unable to afford essential materials for delivery.

.

*Some don’t have money, and some are even struggling with poverty and hunger. A lot of people who give birth at my place do not even have baby clothes. I have to take my wrapper to wrap the baby in it since I don’t have a small baby as my last born is 16 years now and since* I *don’t have any clothes to give her, I had to tear my wrapper to wrap the baby with it (****Older Women’s Group, Kaduna State****).*

Many FGD participants noted the fear of contracting COVID-19 in the healthcare setting as a barrier to the use of facility-based SRH services during the COVID-19 lockdown. Some of the women expressed the fear of being tested for COVID-19 and being found to be positive for infection when they visit health facilities. This group of participants expressed the fear that testing positive for COVID-19 would lead to hospitalisation and seclusion from their family members. Others were worried about contracting COVID-19 from the health facility if they attend services there.

*Because it was during the COVID time, people were not really working, some people were scared of contracting COVID-19****(Adolescents’ Group, Lagos State).***

*You know during that pandemic everybody was scared especially of going to the hospital because if you are having another issue when you get to the hospital you are scared of what they tell you you are having****(Older Women’s Group Lagos State).***

The attitude of the health workers was also identified as a barrier to services, particularly concerning family planning. Unmarried women, especially adolescents, who had sought contraceptives reported being discriminated against by health service providers, and in some cases faced stigmatisation by the health workers. Another factor relating to the healthcare settings that constituted a barrier to accessing care was the impersonal attitude demonstrated to clients and patients by health workers, resulting from the need of the health workers to protect themselves from being infected by COVID-19 through their clients and patients. The sense of health workers being “distant” from the patients and clients was probably further accentuated by the use of coverall personal protective devices.

*It is different because before COVID-19, access to health care was very easy. When there was nothing like COVID-19, the doctors and nurses were always in the hospital, they meet with you. The nurses take your complaints and give them to the doctor without any issue of whether you have COVID-19 or not. But during COVID-19 it wasn’t like that! Before the nurse attends to you, they will want to cover everywhere as if you’re having leprosy so it was very scary. (****Older Women’s Group, Akwa Ibom****)*

### Alternative sources to orthodox health facilities for SRH care during the COVID-19 lockdown

**Fig. 2:**
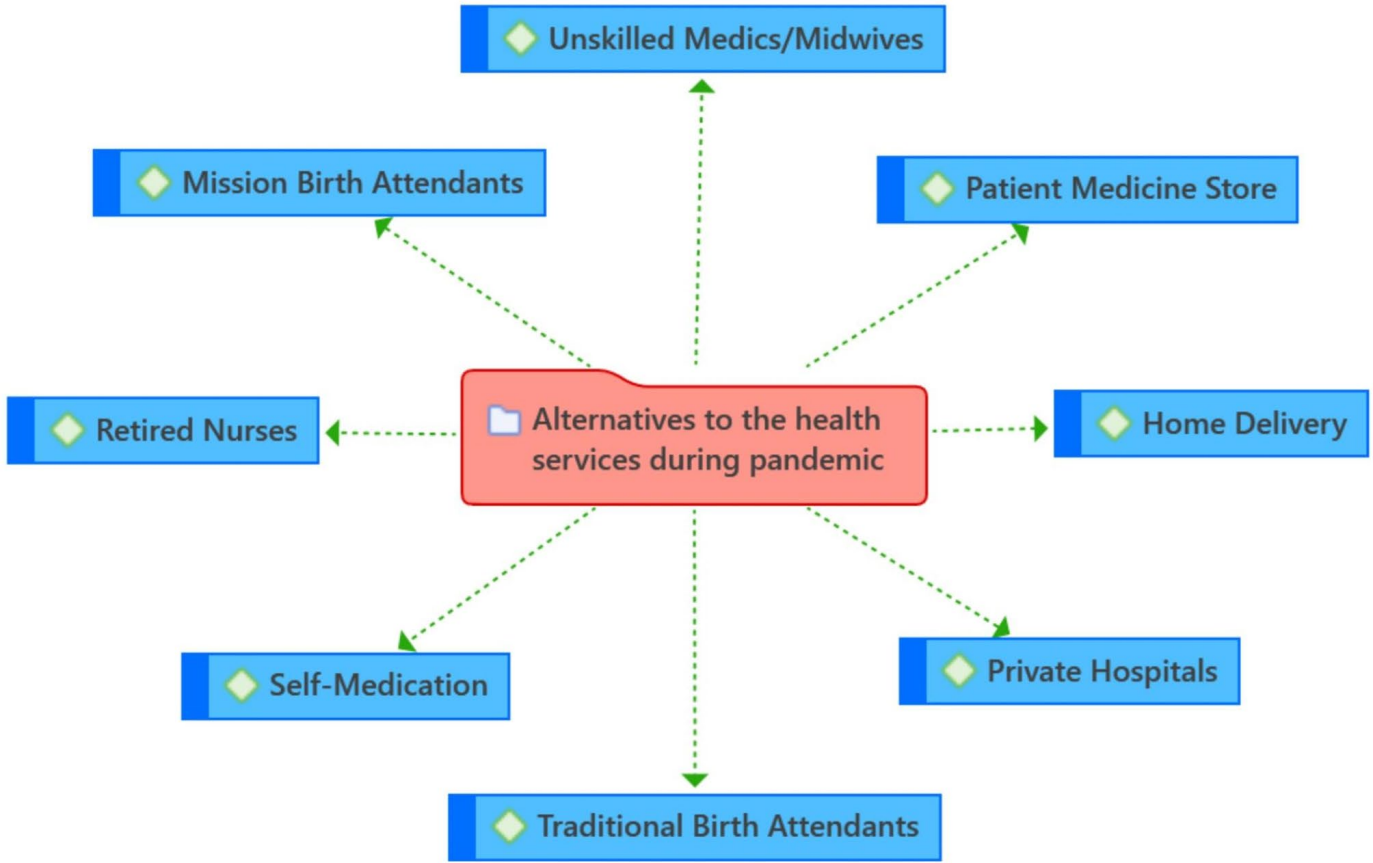
Alternatives sources to public sector health facilities used by women of reproductive age for SRH services during the COVID-19 lockdown

FGD participants reported that in the face of challenges with the availability and accessibility of services in public-sector health facilities during the COVID-19 lockdown, women sought SRH care from several other alternative sources– both orthodox and non-orthodox healthcare sources (Fig. 2).

The opinions of the FGD participants strongly suggested a significant rise in the use of non-orthodox sources, particularly for those that had challenges with geographical and/or financial access to orthodox health services.

*Since the means (i.e. funds) of going to the hospital or clinic are not available at that moment, some may result to self-medication or they will, like it is said before, they will ask another woman or those that are already experienced that aspect (of health problem) that, “what did you do when you had a similar situation in your pregnancy.” They might rightly tell them …although they know that the person is not a medical practitioner but she needs to be well. So, she can go in her own way resulting in self-medication. (****Adolescents’ Group, Abuja).***

The FGD participants opined that the need to seek alternative healthcare sources outside public sector facilities appears to be greatest in the case of pregnancy-related services. Broadly, women requiring ante-natal and delivery services resorted to five groups of alternative healthcare sources outside the public sector facilities: (i) skilled attendants in other healthcare settings (private health facilities) or community settings (such as retired midwives within the community); (ii) non-skilled attendants but formally licensed groups, particularly proprietary and patent medicine vendors (PPMVs); (iii) unlicensed groups with an unknown level of competence who are often referred to as “local” nurses and midwives; (iv) community-based resource persons, including traditional birth attendants, herbal practitioners, older women experienced in childbirth; and (v) self-help, including self-medication with either or both orthodox and herbal preparations, and other forms of home-based care.

The use of traditional birth attendants (TBA) for child delivery services was reported to have increased in many settings. In the absence of facilities and services, older women of the community volunteered to provide childbirth services at home. This was particularly reported in Kaduna and Akwa Ibom States. Some of the TBAs, however, noted that they referred pregnant women to the facilities in situations when there were complications. An older woman in Akwa Ibom State narrated her experience when she had to be involved in a home delivery as a result of the lockdown as follows;

*The experience I had with one pregnant woman before she could come to the hospital was that she was in labour that day. She was calling “nurse o, nurse o, nurse o”. But there was nobody that could come out. She was shouting, “come and hold my baby for me o.” Because my house is close, so I had to rush. She was still shouting, “please o, help me, please help me.” Before we know it, she put to bed outside. She gave birth to a baby girl, and I ran to my place and picked up some few things and went to call one woman that live close to us also, I had to call that patient medicine vendor woman and Eka Mandu (Mandu’s mother). So, three of us succeeded in cleaning her up and take her home****(Older Women’s Group, Akwa Ibom State)***

*You know during that pandemic everybody was scared especially of going to the hospital because if you are having another issue when you get to the hospital you are scared of what they tell you, you are having. So, that means even if you are having headache, they would tell you it is another thing. So, people fear going to health centre or hospital; they prefer taking herbs.****(Older Women’s Group, Lagos State).***

### SRH service gaps during COVID-19 lockdown and its effect on women’s SRH situations

The effectiveness of SRH care supply in terms of how well it aligns with the need of women during the COVID-19 lockdown was reported to be low. The consequent gaps between the healthcare supply and the needs impacted negatively on SRH and the well-being of women across the study sites. The majority of our FGD participants expressed the opinion that there was an increase in the number of women who experienced domestic violence in their communities and there was no effective response to their needs by the available SRH services. The participants also noted that there was an increase in the rates of sexual activities, particularly among unmarried adolescents. Factors associated with the increased sexual activities, according to the FGD participants, include boredom and idleness associated with the closure of schools, recreational, and other public facilities. The desire of adolescent girls to meet their financial needs was reported to have led to sexual partnerships with older men who could respond favourably to their needs. However, with challenges in contraceptive supply and accessibility, an increased incidence of unplanned pregnancy was reported in most of the FGD sessions. There were also reports of an increased level of early marriage, as many unmarried but pregnant adolescents chose marriage to avoid the shame and stigma associated with an out-of-wedlock pregnancy.

*Some of us are forced to have boyfriends when we are not supposed to have. And some of us are also forced to go and have ‘sugar daddies’ (older male sexual partners who provide financial support) when we are not supposed to have. And you see that these things affect us. During the COVID-19 lockdown, we didn’t go to school. And that affected some of us further…. You see some girls, maybe 15 years, getting married and then going ahead to have an issue when she is not supposed to****(Adolescents’ Group, Akwa Ibom State).***

*Some women missed their dose of (contraceptive) injections. I had a friend who had some side effects of the method she was using, so she stopped and decided to go back to the hospital but she couldn’t due to the immobility. So as a result of this, she got pregnant.****(Older Women’s Group, Borno State)***

*Unwanted pregnancy… you see somebody pregnant. Some they will not even pay anything (i.e. the man does not formally request the hands of the girl in marriage from her family), they will just say, they will move in. they don’t want to have some shame: they will move in and say they are married****(Adolescents’ Group, FCT)***

## Discussion

This study is one of the few qualitative studies to have examined the impact of the COVID-19 lockdown on access to healthcare, and specifically SRH services, in Nigeria. It is also one of the few studies – quantitative or qualitative – that have embraced both the southern and northern regions of Nigeria in examining the effect of COVID-19 on healthcare access in Nigeria’s multi-ethnic setting. Thus, this study provided a broader insight than the previously published qualitative studies [17, 24, 32] and complements the earlier published quantitative studies [14–16, 18] on the effect of the COVID-19 lockdown on SRH services. Our study makes a unique contribution to the existing literature by specifically exploring the alternative healthcare sources used by women for SRH care in the face of constrained availability and accessibility of orthodox healthcare in public sector health facilities. Furthermore, the study provides insight into the choices women made in those circumstances, which may impact have impacted the SRH outcomes.

As our findings show, the access of women to SRH services through public health facilities became reduced during the COVID-19 lockdown and, thus, the utilisation of SRH services during the lockdown reduced despite the greater need for SRH services such as contraception for sexually active populations and care for survivors of gender-based violence. On the one hand, services were less available and, on the other hand, women experienced challenges in reaching the available services. Our findings complement the report of previous quantitative studies in Nigeria: Adelekan et al. reported a 2–6% reduction in utilisation of various SRH services in primary healthcare settings in Nigeria [16] while Balogun and colleagues reported that about a third of the women seeking SRH care in Lagos during the lockdown had a challenge with accessing orthodox services [18]. Results from other settings also show that the Nigerian experience is similar to what obtains in several sub-Saharan African countries and other low- and middle-income settings [12, 23, 33–35].

Factors associated with the availability of SRH services in our study are related primarily to the service context, organisation and resources. Among others, several health facilities had a reduced workforce for service provision at any point in time during the lockdown. The desire to maintain social distancing in healthcare settings, re-assignment of some health workforce for the provision of COVID-19-related services and restriction of vehicular movements contributed to the reduction in the number of staff available to provide SRH services. Respondents in our study also reported the closure of some health facilities during the lockdown, while some other facilities offered less intense services than in the pre-lockdown era. These findings agree with a previous report that while most PHC facilities in Nigeria were opened during the lockdown, about a quarter of them was only offering partial services [18].

Regarding the challenges faced by women in accessing available SRH services during the COVID-19 lockdown, our findings confirmed the role of some previously reported barriers as well as identified additional barriers. Previously reported barriers include difficulties with vehicular availability and movement; the fear of contracting COVID-19 during a visit to the health facility; and the potential or experience of being harassed on the road by law enforcement officers [23, 24]. In addition, we found that the fear of being compelled to take a COVID-19 test when visiting a health facility with the prospect of being taken into an isolation centre if tested positive, negatively impacted care-seeking. The expectation of a long waiting time in health facilities because of the reduction of available healthcare personnel was also a factor. The attitude of health workers was also recorded as a barrier to seeking and accessing care during the COVID-19 pandemics – similar to what has been reported from some other studies [24, 36]. While COVID-19 reinforced some of the existing negative attitudes of health workers, such as the stigmatization of unmarried adolescents seeking contraceptive services [37, 38], the desire for personal safety also appeared to have made health workers more impersonal in the caregiving process during the COVID-19 lockdown. These findings also underscore the importance of acceptability, which is conceptualized by Levesque and colleagues [27] as one of the five dimensions of healthcare accessibility, and framed as the ability to seek care.

The public health sector is the main source of most SRH care for the majority of women in Nigeria, including family planning, maternal care and HIV/AIDS services [39–41], with the cheaper cost and wider spread as key factors. In most of Nigeria’s rural areas, private health facilities are lacking and public sector facilities are the only sources of skilled orthodox care. Compared to the public sector, the proportion of private facilities that provide SRH services like family planning is considerably low [40, 41]. The extant national policy of providing family planning services and treatment of HIV free of charge to clients in public health facilities also increases the preference of many women for SRH services in public sector facilities. Furthermore, several state governments provide pregnancy-related care and child delivery services free of charge in their health facilities. Thus, women using the public sector facilities for SRH would likely have faced a significant dilemma when they had to find an alternative source of care during the COVID-19 pandemic and lockdown situation and were confronted with both geographic and financial access issues.

Our findings show that in the face of challenges with accessing SRH care in public sector facilities, women did not readily turn to private health facilities where skilled orthodox health care may also be available. Rather, the alternative healthcare sources that were mostly used by women included informal community-based resources/services such as retired healthcare workers, TBAs, and PPMVs. A high level of self-care and self-medication with orthodox and non-orthodox medicine was also reported. Similar findings of a shift to locally available services, including TBAs, PPMVs, and home remedies have similarly been reported in several African countries, particularly in slum communities [42]. A Nigerian study has also reported a significant increase in patronage of medicine vendors in South-East Nigeria during the lockdown [43]. Our findings also showed that many women sought healthcare help from neighbours with no medical training background but who have had some personal experiences with certain health situations. Among others, older women who are not formally recognized as TBAs attended to women in labour in many communities across Nigeria. However, a study in India [44] reported that participants found private care to be more competent than public healthcare during the COVID-19 lockdown.

Given the choices reported to have been made by women as alternative sources for healthcare during the COVID-19 lockdown in our study, they did not appear to have prioritized levels of healthcare skills. Neither did the women show a strong preference for other facilities offering orthodox health care; otherwise, the private health facilities would have been the preferred alternate source of care to orthodox public health facilities. A major factor that appeared to have driven the women’s choices is geographic accessibility: the nearness, ease and ability to reach the healthcare source and readily receive help. The nature of the facilities – whether formal or informal sources, orthodox or non-orthodox – did not matter as much. The women’s choices in this respect may have been constrained by the restriction of movement during the COVID-19 lockdown and the unpredictable timing of SHR events such as labour and childbirth. The fear of going to health facilities with the potential to contract COVID-19 or be compelled to take the COVID-19 test, as earlier discussed, may also have played a role.

Financial accessibility played a major role in women’s choices of alternative healthcare sources. In this respect, the financial situation of the women and their households at the time of the lockdown where economic activities were at a very low level and the affordability of the healthcare cost may have constrained the women’s choices. The potential financial cost, for example, may have been a major factor in the non-popularity of orthodox private health facilities – which are generally more expensive than the public sector and informal healthcare sector – as an alternative source when public sector facilities were not available or readily accessible. As noted earlier, the Nigerian government mandates the provision of several SRH components in public-sector facilities free of direct cost to clients. Tthus, switching to relatively costly alternative sources of SRH care during the COVID-19 lockdown may have been difficult for most women who had been patronising public facilities. Thus, the financial or economic factors could have accentuated the adoption of self-medication, use of non-orthodox care, seeking help from non-medical neighbours, and patronage of TBAs and drug vendors. The importance of financial accessibility to healthcare and the role of financial affordability of healthcare in healthcare decision-making and care-seeking behaviour have severally been discussed in the literature [25, 45].

Our findings also highlight the high cost of the gap between SRH service needs and supply that results from decreased availability and accessibility of SRH services during the COVID-19 lockdown, particularly in terms of unplanned pregnancy. In particular, given the scenario of increased adolescent sexual activities during the lockdown and discrimination of health workers against unmarried adolescents seeking contraceptive services, an increase in unplanned pregnancies is a natural consequence. Our study also reported an increase in early marriage associated largely with unplanned pregnancies. Musa and colleagues in a recent commentary have described early marriage and teenage pregnancy as “unspoken consequences of COVID-19 pandemic in Nigeria” [46].

Nigeria’s restricted law on abortion and difficulty in accessing safe abortion services, and the stigma associated with out-of-wedlock pregnancy provide some contexts for an increase in early marriage. Nigeria’s law only permits the termination of pregnancy in circumstances where the continuation of such pregnancy threatens the life of the mother [47]. The law stipulates severe punishment for all individuals involved in any abortion that is performed on other grounds, including the woman on whom abortion is performed as well as the person performing the act (Sects. 228–230 of the Criminal Code Act and Sects. 232–233 of the Penal Code) [48]. Adolescent girls with unplanned pregnancies and their families most likely considered marriage as a face-saving path to avoiding the shame and stigma of out-of-wedlock pregnancies as access to safe abortion was challenging. Our findings also corroborate the association between the COVID-19 lockdown and an increase in the incidence of SGBV in Nigeria as previously reported in other studies [48, 49].

## Strengths and limitation

With a study setting spanning two northern and two southern states and the Federal Capital Territory, we were able to capture the experiences of women in diverse geographies, which is important in Nigeria’s multi-ethnic setting. Also, our study participants included both adolescent and older women, as well as married and unmarried, thereby enabling us to capture the different SRH and care-seeking experiences that may differ by sociodemographic characteristics. Thus, our study provides a better overview of the experience of women during the COVID-19 lockdown in Nigeria compared to previous qualitative studies. We purposely chose women with considerable knowledge of their community, who had lived in the community through the lockdown period, and knowledgeable of the SRH care-seeking experiences of women during the lockdown to provide the data needed to meet our study objectives. Furthermore, we adopted a holistic perspective of SRH and our inquiry broadly covers various components of essential SRH services. Overall, the framing of the study and the approach adopted enabled us to gain insight into the alternative healthcare sources used by women when faced with significant barriers to accessing SRH care from public health facilities in Nigeria’s pluralistic health system.

However, as with all qualitative studies, our study involved only a small sample of participants and participants were purposely selected; as such, our findings are not intended to be quantified or generalized. Also, there is the possibility of some level of social desirability bias in the women’s responses, but the leaning of the responses towards the use of non-orthodox and non-skilled care rather than the more acceptable orthodox care reflects that the likelihood of the bias is low.

## Conclusion

COVID-19 has impacted the health system considerably, and the associated lockdown particularly negatively affected the availability as well as the accessibility of SRH services. The COVID-19 lockdown exacerbated pre-existing barriers to healthcare access as well as led to the emergence of new barriers. To overcome the challenges posed by these barriers and obtain the SRH services they desire, women opted for various alternatives, consisting of both orthodox and non-orthodox traditional forms of care as well as utilized community resources and self-help approaches. These alternatives, particularly the non-orthodox approach and the use of non-skilled health workers, have significant implications for the effectiveness of the services received and the overall health and well-being of women.

With the preference of women for locally available services when access to formal health services became constrained, community systems strengthening is critical to overcoming barriers such as may be experienced during lockdowns. Among others, a robust system for health at the community level is imperative for the future, including effective community-based health services, appropriately empowered community resource persons, and health-literate individuals and households. It is also crucial to identify key resource persons and stakeholders in the community who would champion the cause of SRH services in the event of emergencies and health security situations. Such persons could serve as a liaison between the formal health system and community system and provide local guidance in the event of emergencies. Overall, the reports of increased sexual engagement among adolescent girls, unplanned pregnancy, early marriage, and SGBV in this study point not only to the need for continuity of quality SRH services but also underscore the need for a higher supply of SRH services during periods of lockdown occasioned by disease outbreaks.

## Data Availability

All data generated or analysed during this study are included in this published article [and its supplementary information files].

## List of Abbreviations

COVID-19: Novel Human Coronavirus Disease 2019
FCT: Federal Capital Territory
FGD: Focus Group Discussion
FP: Family Planning
PHC: Primary Health Centre
PPMVs: Patent and Proprietary Medicine Vendors
SGBV: Sexual and Gender-Based Violence
SRH: Sexual and Reproductive Health
TBA: Traditional Birth Attendant
